# Circular RNA P4HB promotes glycolysis and tumor progression by binding with PKM2 in lung adenocarcinoma

**DOI:** 10.1186/s12931-023-02563-7

**Published:** 2023-10-25

**Authors:** Haoran Li, Haifa Guo, Qi Huang, Shaodong Wang, Xiao Li, Mantang Qiu

**Affiliations:** 1 Department of Thoracic Surgery, Peking University People’s Hospital, No. 11 Xizhimen South Street, Beijing, 100044 China; 2 Thoracic Oncology Institute, Peking University People’s Hospital, Beijing, 100044 China; 3 The First Department of Thoracic Surgery, Beijing Chest Hospital, Capital Medical University, Beijing, 101149 China; 4 Department of Thoracic Surgery, The First Affiliated Hospital of Zhengzhou University, Zhengzhou, 450003 China

**Keywords:** Lung adenocarcinoma, circP4HB, PKM2, Macrophage polarization, Aerobic glycolysis, Prognosis

## Abstract

**Background:**

Emerging evidence indicates that circular RNAs (circRNAs) play vital roles in tumor progression, including lung adenocarcinomas (LUAD). However, the mechanisms by which circRNAs promote the progression of LUAD still require further investigation.

**Methods:**

Quantitative real-time PCR was performed to detect the expression of circP4HB in LUAD tissues and cells. Then, Kaplan–Meier analysis was used to determine the prognostic value of circP4HB expression. We employed RNA pull-down, RNA immunoprecipitation, mass spectrometry, cells fraction, glucose consumption, lactate production, pyruvate kinase M2 (PKM2) activity, and macrophage polarization assays to uncover the underlying mechanisms of circP4HB in LUAD.

**Results:**

We found that circP4HB is upregulated in LUAD tissues and correlated with advanced TNM stages and lymph node metastasis. LUAD patients with high circP4HB expression had poor prognoses. Functionally, circP4HB promoted LUAD progression in vivo and in vitro. Upregulated circP4HB increased glucose consumption, lactate production and accelerated aerobic glycolysis in LUAD cells. Mechanically, circP4HB mainly accumulated in the cytoplasm of LUAD cells and bound with PKM2 and subsequently upregulating PKM2 enzymatic activity by increasing its tetramer formation. Additionally, circP4HB promoted M2 macrophage phenotype shift via targeting PKM2. Finally, rescue assays further confirmed that circP4HB could promote LUAD cell progression through its interaction with PKM2.

**Conclusion:**

These results demonstrate that circP4HB could promote LUAD progression, indicating circP4HB might be a potential therapeutic target of LUAD.

**Graphical Abstract:**

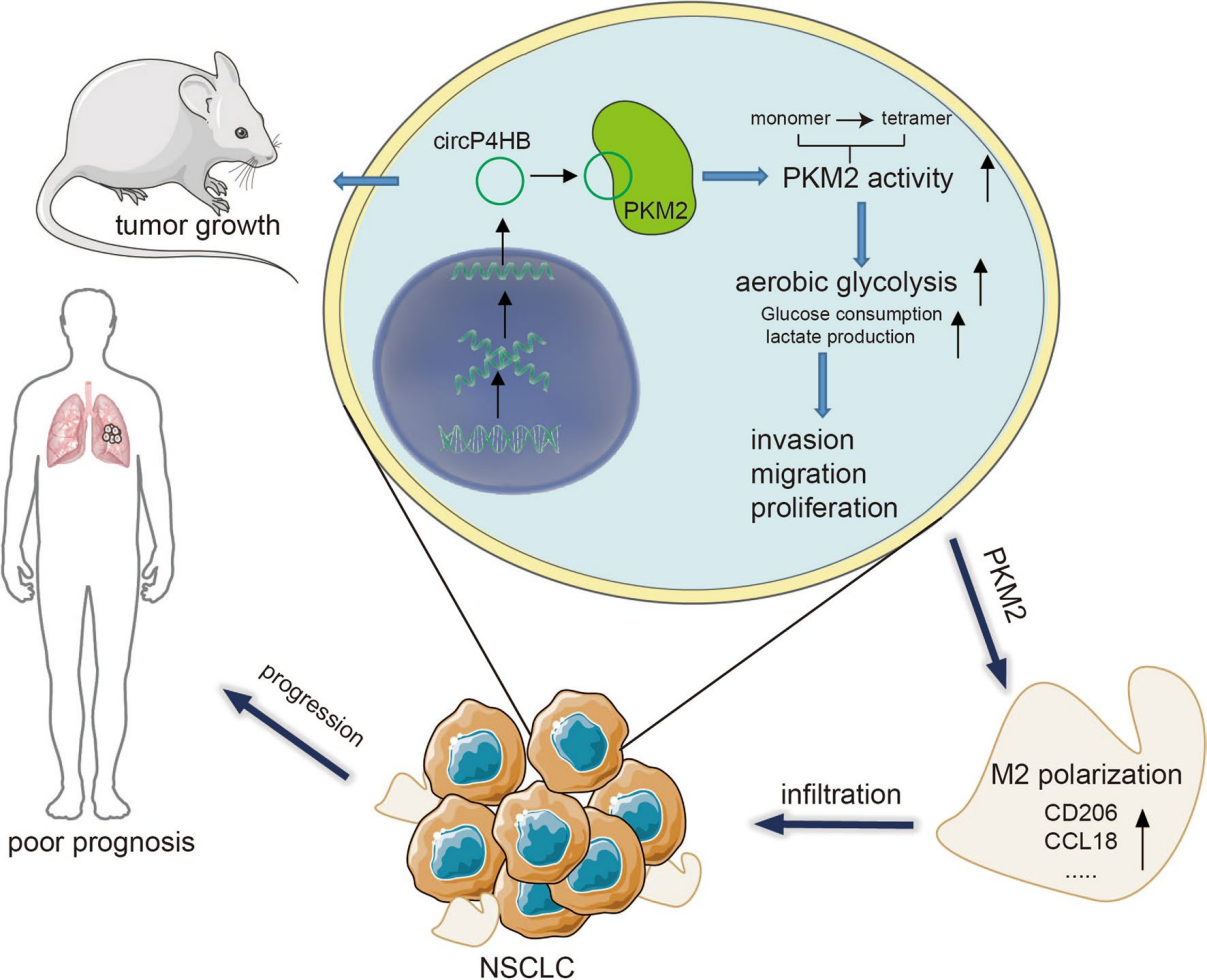

**Supplementary Information:**

The online version contains supplementary material available at 10.1186/s12931-023-02563-7.

## Introduction

Lung cancer (LC) remains a leading cause of cancer-related mortality in both China and the USA, with non-small cell lung cancer (NSCLC) accounting for approximately 85% of cases [1]. Among NSCLC subtypes, lung adenocarcinoma (LUAD) represents about 50% of cases [2]. Despite significant advancements in therapeutic approaches, including targeted therapy, immunological treatments, and the discovery of novel biomarkers, the prognosis for LUAD patients remains poor [3–6]. Thus, deep understanding of mechanisms behind LUAD progression and identification of reliable biomarkers for LUAD are urgently required.

CircRNAs are covalently closed structures without 5’-caps and 3’ polyA tails so that they are resistant to RNase R enzyme [7]. CircRNAs participate in various biological processes in vivo like cell proliferation, apoptosis, cell differentiation and cancer development [8]. CircRNAs exert their function as ceRNA or miRNA sponges, modulating alternative splicing or transcription, interacting with RNA binding proteins (RBP), and even directly translating proteins [9]. For example, circPRKCI can bind to both miR-545 and miR-589 and inhibits their suppressing capability on E2F7, subsequently promoting LUAD progression [10]. CircXPO1 promoted LUAD development by binding to IGF2BP1 and stabilizing CTNNB1 mRNA [11]. Nonetheless, the underlying mechanisms of circRNAs facilitating the LUAD progression remain largely unknown.

Aerobic glycolysis, also called the “Warberg effect”, refers to cancer cells performing glycolysis in the cytosol even in the presence of adequate oxygen. Cancer cells grow fast than normal cells and the production of adenosine triphosphate (ATP) by glycolysis is much faster than oxidative phosphorylation in the mitochondria [12]. Moreover, aerobic glycolysis provides critical intermediate metabolites for cancer growth, for instance, Fascin can promote glycolytic flux via upregulating the expression and activities of phosphofructose-kinases 1 and 2 [13]. Previous studies also showed circRNAs can promote tumor development by regulating the key enzymes of glycolysis [14, 15]. But, the relationship between circRNAs and glycolysis needs to be further explored.

Herein, we identified a novel circRNA, circP4HB, from the P4HB gene, that can promote LUAD progression, accelerate aerobic glycolysis and induce M2 macrophage polarization by interacting with pyruvate kinase M2 isoform (PKM2). Therefore, circP4HB may be a reliable biomarker for the prognosis and progression of LUAD.

## Materials and methods

### Patients and samples

Ninety-eight LUAD tissues and paired adjacent normal tissues were obtained from patients who underwent surgery in the Department of Thoracic Surgery, Peking University People’s Hospital. The CNV of P4HB was initially evaluated in twenty-four LUAD tissues along with their paired adjacent normal tissues and then confirmed in an additional sixty-one LUAD tissues and their paired adjacent normal tissues. None of the patients included in this study underwent any preoperative radiation or chemotherapy. The clinical and follow-up information was collected to further analyze. The pathological analysis was based on the 8th TNM classification of IASLC. Written informed consent was obtained from each patient. This study was approved by the Ethical Review Committee of Peking University People’s Hospital.

### Cell lines and cell culture

The human LUAD cells (A549, H1299, SPCA1), THP1 and 16HBE (a normal human bronchial epithelial cell) were purchased by the Cell Bank of Chinese Academy of Sciences. A549, H1299 and THP1 were cultivated in RPMI-1640 (Gibco, life technologies, California, USA), while SPCA1 and 16HBE were maintained in DMEM (Gibco, life technologies, California, USA). All media were supplemented with 10% fetal bovine serum (FBS; Gibco, life technologies, California, USA) and 1% streptomycin plus penicillin (Biosharp, Anhui, China). The incubation atmosphere was at 37 °C in a humidified incubator with 5% CO_2_.

### RNA extraction and qRT-PCR

Total RNA was extracted from tissues and cells using Trizol (Invitrogen, Carlsbad, CA, USA) according to the user’s instructions. Then, RNA was converted into cDNA by PrimeScript™ RT Master Mix (TaKaRa, Shiga, Japan). Quantification of RNA (circRNA and mRNA) was carried out using PowerUp SYBR Green Master Mix (Thermo Fisher Scientific, Waltham, MA, USA). Each quantitative polymerase chain reaction was performed in the BioRad CFX96 Sequence Detection System with a total reaction volume of 10 μl (BioRad company, Berkeley, CA). The gene GAPDH was used as an internal control. All experiments were performed in triplicate and the results were converted into the fold change. The primers included in this study were shown in Additional file 1: Table S1.

### siRNAs, overexpression plasmid and cell transfection

Specific siRNAs or plasmids were used to silence or overexpress circP4HB and PKM2, respectively. The siRNAs targeting circP4HB or PKM2 were designed and synthesized by GenePharma (Shanghai, China). Moreover, circP4HB-overexpressing plasmids were synthesized and purchased by GENERAY Bio-TECH (Shanghai, China). The siRNAs and overexpression plasmids were transfected into cells by Lipofectamine^®^ 3000 (Thermo Fisher Scientific, Waltham, MA, USA) according to the manufacturer’s protocol. The efficiency of silence or overexpression was investigated after 24–48 h of transfection of qRT-PCR or western blot analysis. The sequences of all siRNAs included in this study were listed in Additional file 1: Table S1.

### Proliferation, invasion and migration

Cell proliferation was evaluated by Cell Counting Kit-8 (CCK8, Biosharp, Anhui, China) and colony formation assay. Matrigel invasion was tested in 24-well transwell plates (8-μm pore size, Corning, NY, USA). Forty thousand cells were seeded into each well which had been pre-coated with 100 μl diluted Matrigel (1:7). DMEM or RPMI1640 with 10% FBS was added into low chambers while the serum-free medium was added into upper ones. Then, the cells were fixed with 4% paraformaldehyde and stained with 0.5% crystal violet after incubation for about 48 h. Finally, the cells were observed under a microscope. Cell migration was assessed by wound healing assay as described previously [11].

### Nuclear-cytoplasmic fractionation

Nuclear and cytoplasmic fractions of A549 cells were separated with a PARIS Kit according to the manufacturer’s protocol (Ambion, AM1921). The analysis of RNA was conducted by qRT-PCR. The markers for the nucleus and cytoplasm were respectively U1 and GAPDH.

### MS2-MBP-mediated RNA pull-down and RNA immunoprecipitation assay

MS2-MBP-mediated pull-down was performed as described previously [11]. Briefly, A549 cells were transfected with 50 μg MS2-tagged circP4HB constructs, and 1 × 10^7^ cells were used for each RNA pull-down assay. Cells were harvested 48 h post-transfection and subjected to the RNA pull-down assay to get the potential RNA binding proteins. RNA immunoprecipitation assays (RIP) were performed by a Magna RIP RNA-Binding Protein Immunoprecipitation Kit (Millipore, USA) according to the manufacturer’s instructions.

### Nucleic acid electrophoresis

The cDNA and PCR products were analyzed by 2% agarose gel electrophoresis with TAE running buffer. DNA was separated by electrophoresis at 120 V for 30 min. The bands were visualized by UV irradiation.

### Western blot

Total protein was extracted from cells with Cell lysis buffer for Western and IP (Beyotime, Shanghai, China) and Protein Phosphatase Inhibitor (Solarbio, Beijing, China). The protein concentration was determined by BCA method. Protein samples were loaded on SDS-PAGE gels and subjected to electrophoresis. After gel separation, the proteins were transferred to a polyvinylidene fluoride (PVDF) membrane. Then, membranes were blocked in 5% BSA in Tris-buffered saline-Tween 20 (TBST) for 1 h at room temperature and subsequently incubated with primary antibodies overnight at 4 °C. Membranes were washed three times with TBST and incubated with the corresponding HRP-conjugated secondary antibodies for 1 h at room temperature. Protein detection was performed using a chemiluminescence system (Bio-Rad, USA). The primary antibodies for Western blotting were PKM2 (Proteintech, 15822-1-AP), p-PKM2 (CST, #3827), GAPDH (Proteintech, 10494-1-AP), and β-actin (Proteintech, 20536-1-AP).

### Measurement of PKM2 activity, glucose and lactate

The activity of PKM2 was measured by Pyruvate Kinase (PK) Activity Assay Kit (Solarbio, Beijing, China) according to the instruction. The uptake of glucose and production of lactate were examined using the Glucose Assay Kit (Solarbio, Beijing, China) and CheKine™ Micro Lactate Assay Kit (Abbkine, Wuhan, China).

### Tumor xenograft

Female BALB/c nude mice (5 weeks, 18–22 g) were purchased from Charles River (Beijing, China) and maintained under specific pathogen-free conditions. Mice were randomly assigned to negative control and si-circP4HB or EV control and circP4HB-ov groups. A549 cells transfected with control vector (EV), circP4HB-ov and SPC-A1 cells transfected with negative control siRNA (NC) and si-circP4HB were collected and re-suspend in PBS. For the tumor formation assay, 1 × 10^6^ cells/mouse were subcutaneously injected into one flank of each mouse. Tumor growth was inspected weekly using a straightedge, and the tumor volume was calculated using the standard equation V = 0.5 × D × d^2^ (V, volume; D, longitudinal diameter; d, transverse diameter). The protocol used for these studies was approved by the Animal Care Committee of Peking University People’s Hospital. Moreover, the animal study was carried out according to the State Food and Drug Administration of China’s regulations on animal care.

### M2 macrophage polarization

The THP1 cell line was maintained at 5 × 10^5^ cells/well in the six-well plate with RPMI 1640 medium. Then, THP1 cells were differentiated using 200 nM phorbol 12-myristate 13-acetate (PMA, Sigma-Aldrich) for 3d. Differentiation of PMA-treated cells was enhanced after the initial 3d stimulus by removing the PMA-containing media and then co-cultivating different LUAD cells with the different expression level of circP4HB in fresh RPMI 1640 for 48 h. Finally, the total RNA of THP1 was collected and analyzed by qRT-PCR to assess the expression of markers of M2 macrophage polarization.

### Statistical analysis

Data computation was accomplished by SPSS software 23.0 (SPSS Inc., Chicago, IL, USA). Student’s t-test or one-way ANOVA was applied to determine the significance of differences between two groups or among multiple groups. The Chi-square test or Fisher’s exact test was used to analyze qualitative variables. The strength of the association between continuous variables was analyzed by Spearman correlation analysis. The prognostic value of circP4HB expression was further analyzed using the Kaplan–Meier method. Each experiment was conducted three times. The statistical significance in differences was considered when p < 0.05.

## Results

### The characterization of circP4HB in LUAD

**Fig. 1:**
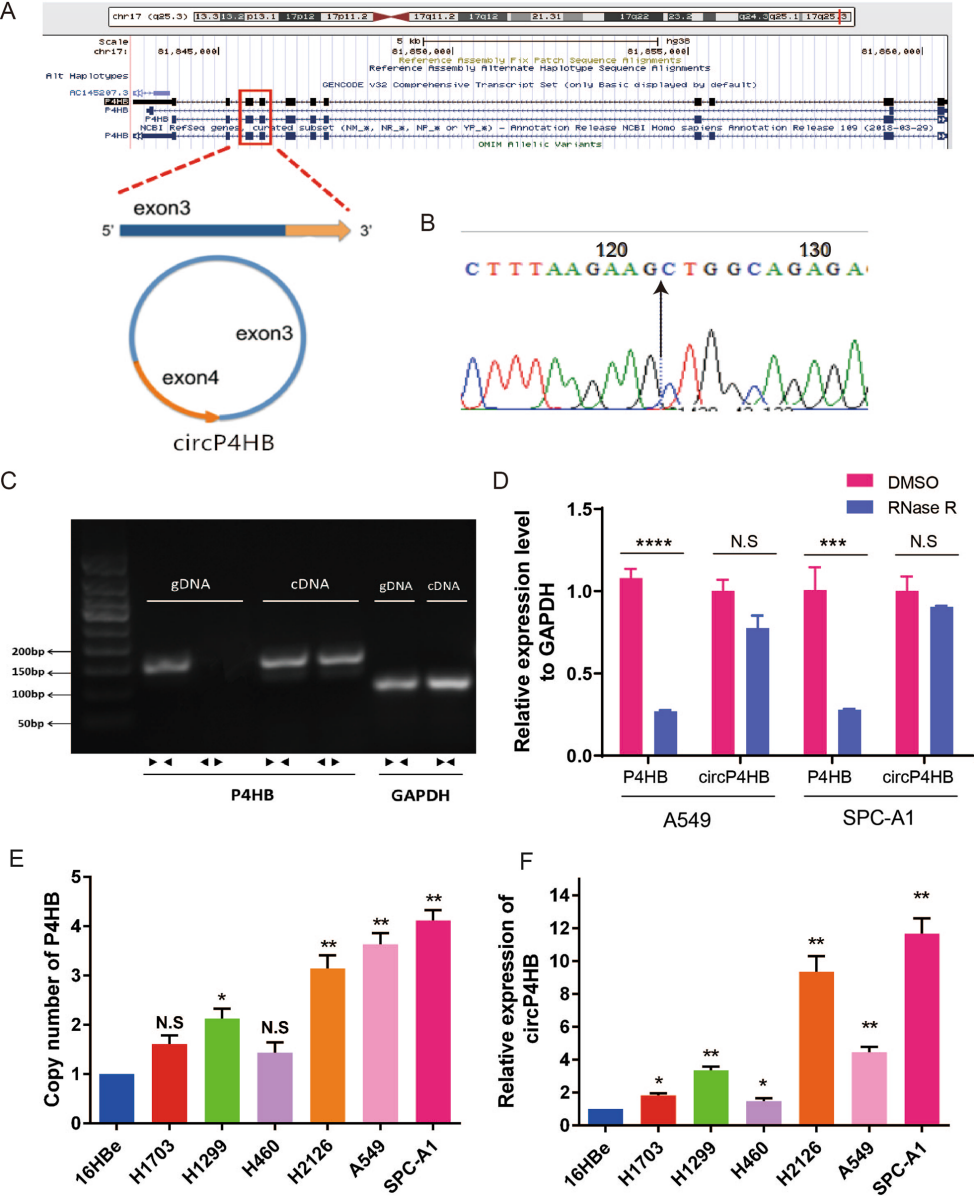
Circular RNA transcript of the P4HB gene. **A** Localization of circP4HB and back-splicing of exon 3 and exon 4. **B** Arrow showed the back-splicing site of circP4HB. **C** Agarose gel electrophoresis analysis of PCR products of divergent primer and convergent primer. ←  → divergent primers; →  ← convergent primers. GAPDH was used as a control. cDNA: complementary DNA, gDNA: genomic DNA. **D** Relative expression level of P4HB and circP4HB in A549 and SPC-A1 cells after RNase R digestion. DMSO treated cells were used as a control. **E** Copy number of P4HB in normal lung cells (16HBe) and lung cancer cell lines. HBE was used as a reference. **F** Expression of circP4HB in normal lung cells (16HBe) and lung cancer cell lines. HBE was used as a reference. (*p < 0.05, **p < 0.01, **p < 0.01, ****p < 0.0001, N.S no significance.)

In our previous work [16], we identified numerous differentially expressed circRNAs in LUAD using ribosomal RNA-depleted RNA sequencing. We also found copy number variation (CNV) of oncogenes may change circRNAs expression. Then, we observed a novel circular transcript of P4HB (circBase ID: hsa_circ_0046263), which is named circP4HB in this work. CircP4HB is a 272-nt circRNA transcript generated by back-splicing of exon 3 and exon 4 of the P4HB gene (Fig. 1A). The result of sanger sequence of PCR product showed the site of back-splicing in Fig. 1B. Divergent primers and convergent primers were designed, and the convergent primers were found to amplify only cDNA, confirming the circular structure (Fig. 1C). Next, the Rnase R treatment assay to further conform the circular structure of circP4HB (Fig. 1D). In addition, both the copy number of P4HB and circP4HB are highly expressed in NSCLC cells compared with 16HBe cells (Fig. 1E, F).

### Correlation between circP4HB expression and clinical characteristics of LUAD

**Fig. 2:**
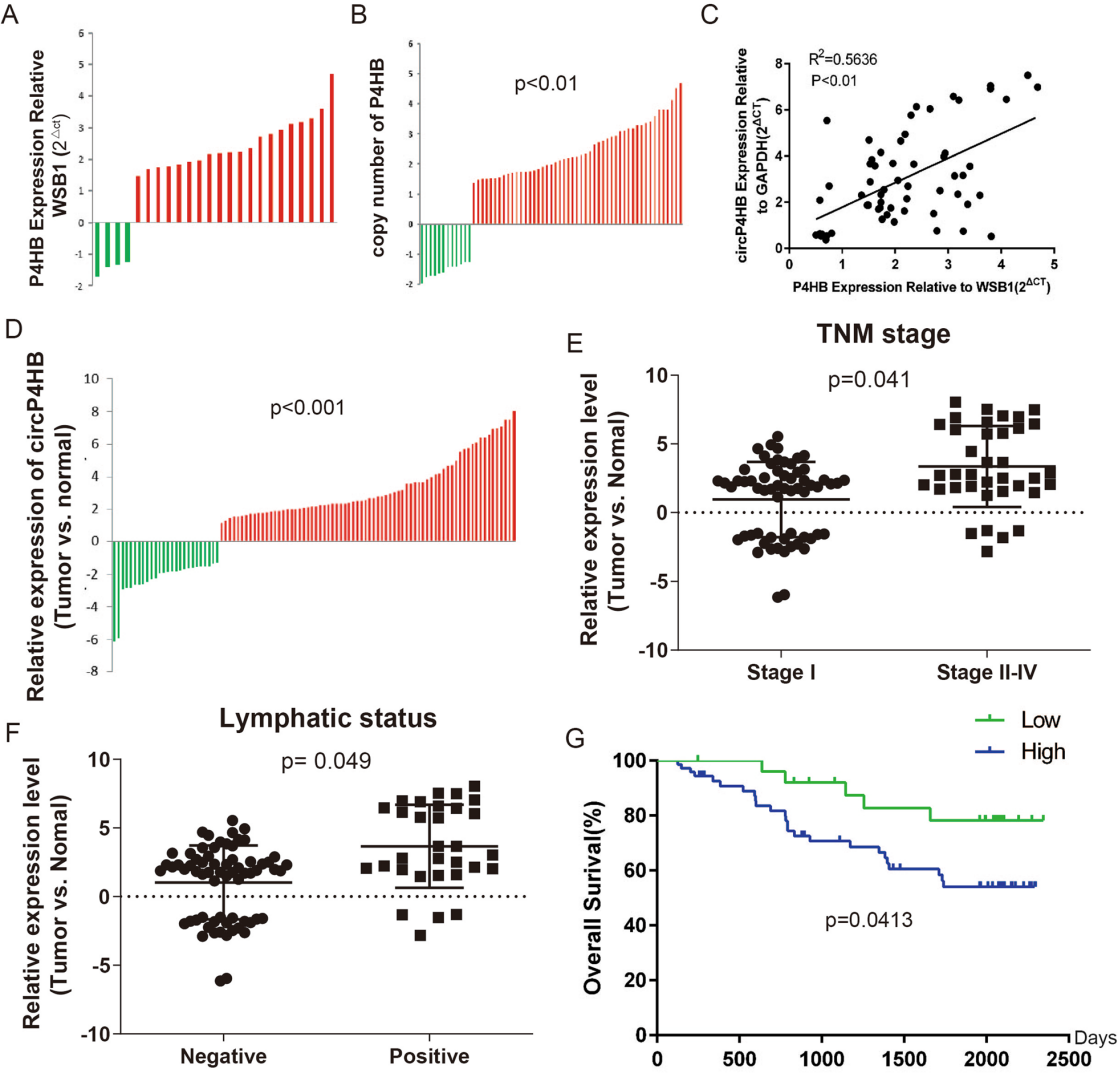
High circP4HB expression is associated with poor prognosis in LUAD. **A** The expression of P4HB gene in LUAD samples, WSB1 was used as a control; **B** Copy number of P4HB in LUAD tissues; **C** Dot plot of P4HB gene copy number and circP4HB gene expression. p-value was calculated by Spearman correlation; **D** the relative expression of circP4HB in clinical samples of LUAD; **E** The circP4HB expression level in patients with tumors different TNM stages (non-paired student test was performed); **F** The circP4HB expression level in patients with lymph node metastasis or not (non-paired student test was performed). **G** Kaplan–Meier analysis of circP4HB in 98 LUAD patients

We first investigated the CNV of P4HB in clinical samples and results showed P4HB is highly expressed in cancerous tissues than in paired normal tissue (Fig. 2A, B). Next, we analyzed the association between the circP4HB level and P4HB gene copy number in LUAD tumor tissues. Intriguingly, the circP4HB expression was a positive correlation with the copy number of P4HB, and this indicated higher circP4HB expression may be caused by the copy number gain of the P4HB gene. Ninety-eight paired LUAD tissues were used to assess the expression of circP4HB and circP4HB is significantly overexpressed in LUAD tissues compared with matched adjacent normal tissues (Fig. 2D). Then, we found circP4HB expression was significantly higher in advanced TNM stage (Fig. 2E) and lymph node metastasis (Fig. 2F). Survival analysis demonstrated that patients with high expression of circP4HB had shorter overall survival times (HR = 2.611, 95%CI: 1.229 to 5.548, p = 0.0413; Fig. 2G), suggesting that high expression of circP4HB might be a biomarker for poor survival of LUAD patients. To further validate the prognostic role of circP4HB, we performed the uni- and muti-variate Cox Regression analysis and the results showed circP4HB was not an independent factor in the LUAD cohort (Additional file 2: Table S2). Taken together, we showed circP4HB is significantly higher expressed in LUAD tissues and higher circP4HB expression of LUAD patients often means a poor prognosis.

### circP4HB promoted proliferation, migration and invasion in LUAD cells

**Fig. 3:**
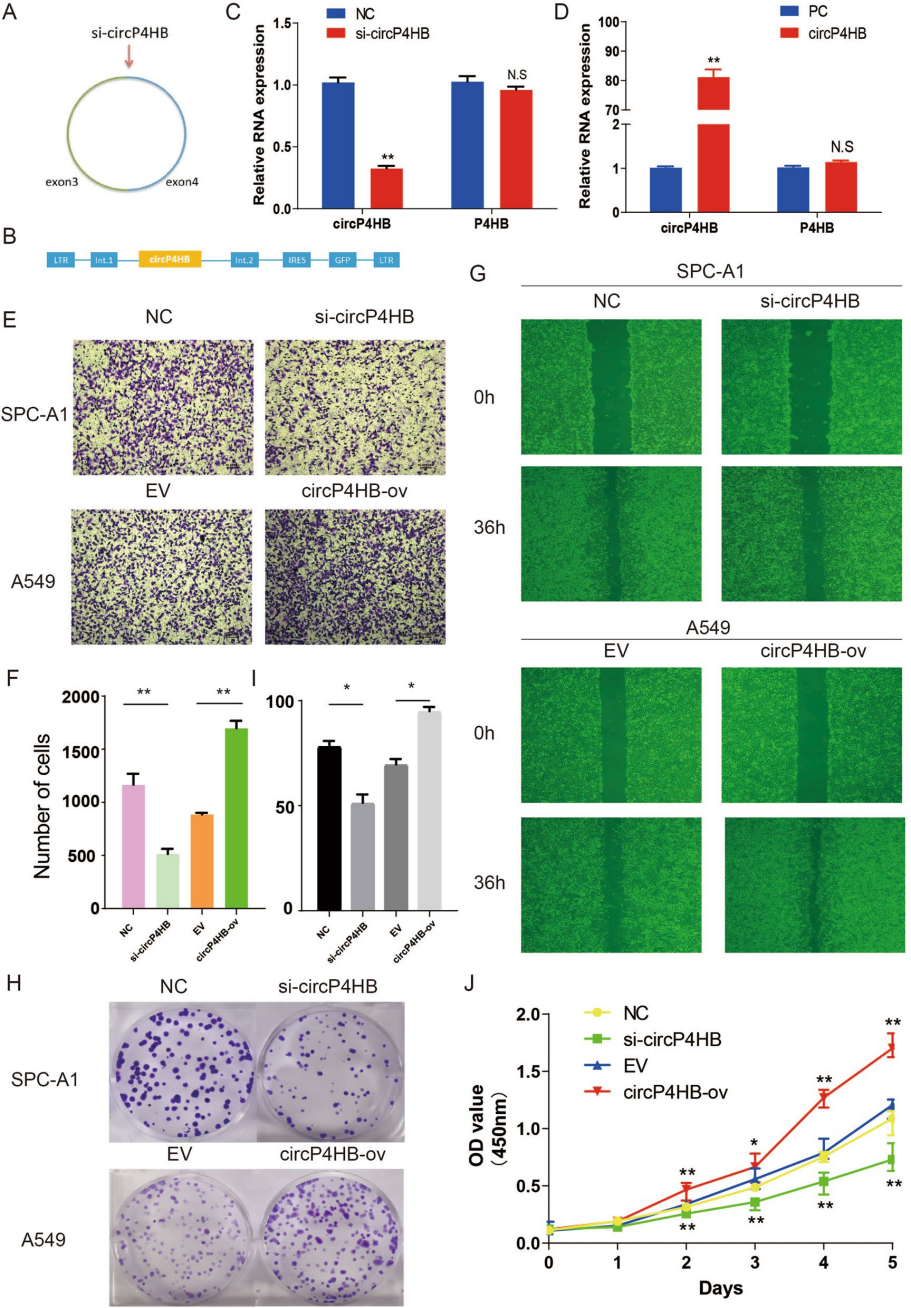
circP4HB promotes LUAD invasion, migration and proliferation in vitro. Schematic illustration showing the siRNA specifically targeting the circP4HB splice junction (**A**) and the overexpression vector (**B**); (**C, D**) showed the efficiency of silencing or overexpressing; (**E, F**) transwell showed the invasion ability of different circP4HB expression; (**G**) wound healing assays illustrated circP4HB accelerate the migration of LUADs; (**H, I, J**) colony formation and CCK8 assays showed circP4HB can increase the proliferation of LUAD cells. (*p < 0.05, **p < 0.01. N.S no significance.)

To investigate the functional role of circP4HB in LUAD progression, we designed a siRNA and an expression plasmid for silencing and overexpressing circP4HB. The siRNA was designed to specifically target the circP4HB back-splice junction site (Fig. 3A) and exon 3 and exon 4 were cloned into an expression vector to overexpress the circP4HB (Fig. 3B). Compared with the negative control siRNA, si-circP4HB greatly decreased the circP4HB level in SPC-A1 cells but did not affect the expression level of linear P4HB (Fig. 3C). Moreover, transfection of the expression vector led to an increase of approximately 80-fold in circP4HB expression compared with that empty vector in A549 cell (Fig. 3D). Moreover, the Rnase R treatment assay to further conform the circular structure of overexpressed circP4HB (Additional file 4: Fig. S1A). Silencing circP4HB significantly decreased in SPCA1 cell but circP4HB overexpression increased the invasion ability in A549 cell (Fig. 3E, F). The wound healing assays showed silencing circP4HB compromised the ability of migration but circP4HB overexpression promoted the migration ability (Fig. 3G, Additional file 4: Fig.S1B). The results of CCK-8 and colony formation demonstrated down-regulated circP4HB significantly suppressed the proliferation ability of SPC-A1 cells, whereas up-regulated circP4HB promoted A549 cell proliferation (Fig. 3H–J). This evidence revealed that circP4HB could promote proliferation, migration and invasion in LUAD cells.

### circP4HB promoted LUAD growth in vivo

**Fig. 4:**
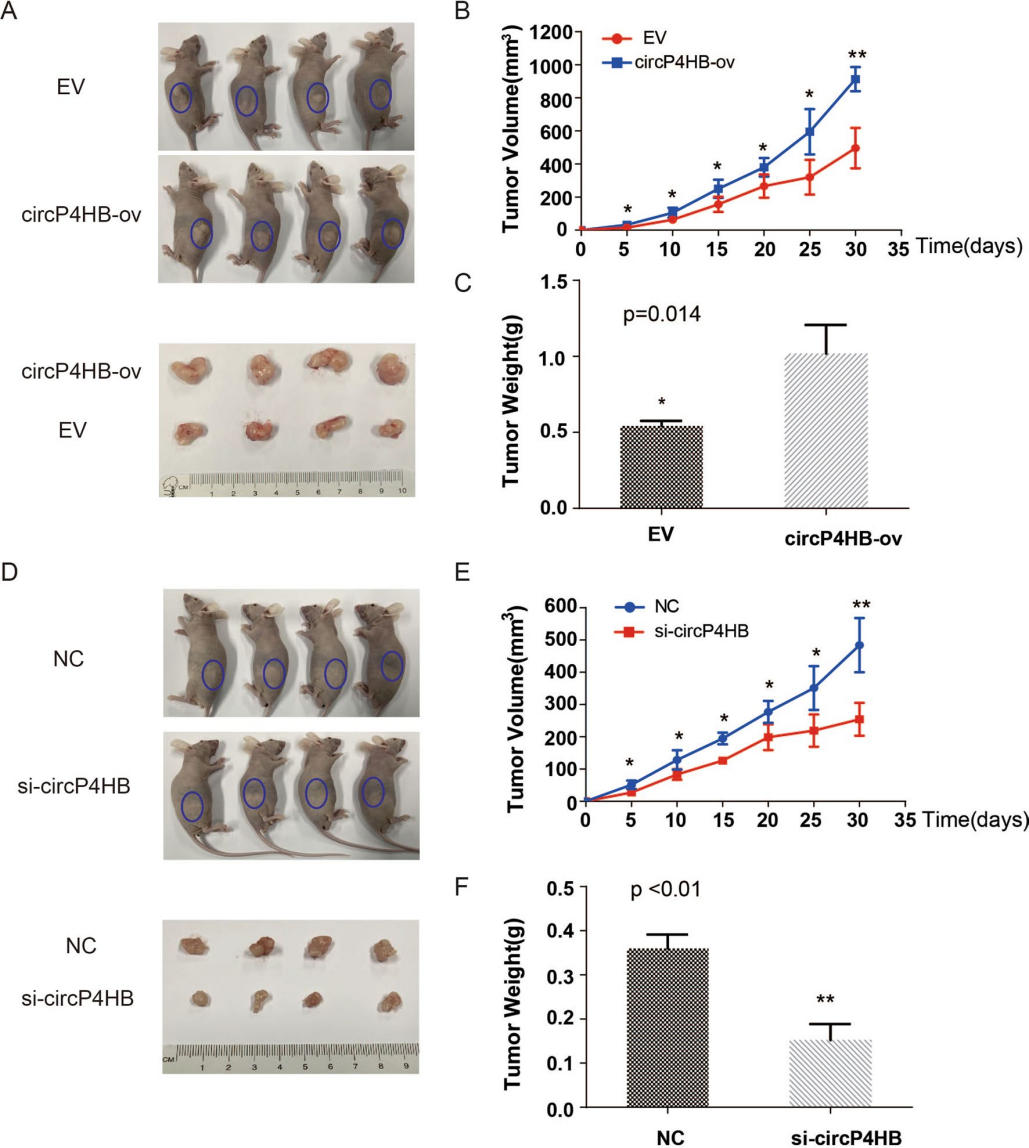
circP4HB promotes LUAD tumor growth in vivo. Xenograft tumor models were established using A549 transfected with control vector (EV), circP4HB-ov and SPC-A1 cells transfected with negative control siRNA (NC) and si-circP4HB. Tumor size (**A**, **D**), growth curve of tumor volume (**B**, **E**), and tumor weight (**C**, **F**) were accessed for each derived xenograft tumor. (*p < 0.05, ** p < 0.01.)

To assess the oncogenic role of circP4HB in vivo, we established xenograft tumor models in nude mice. A549 cells transfected with circP4HB-ov or empty vector (EV) were injected into the right flanks of BALB/c nude mice to generate xenografts. We discovered that circP4HB-ov /A549 injected mice generated larger tumors than EV group (Fig. 4A–C). Then, SPC-A1 cells transfected with si-circP4HB or negative control siRNAs were injected into BALB/c nude mice. The results showed that a significant decrease in tumor volumes and tumor weight from mice injected with si-circP4HB compared with control group was observed (Fig. 4D–F). In summary, circP4HB promoted tumor growth in LUAD in vivo.

### circP4HB can bind to PKM2

**Fig. 5:**
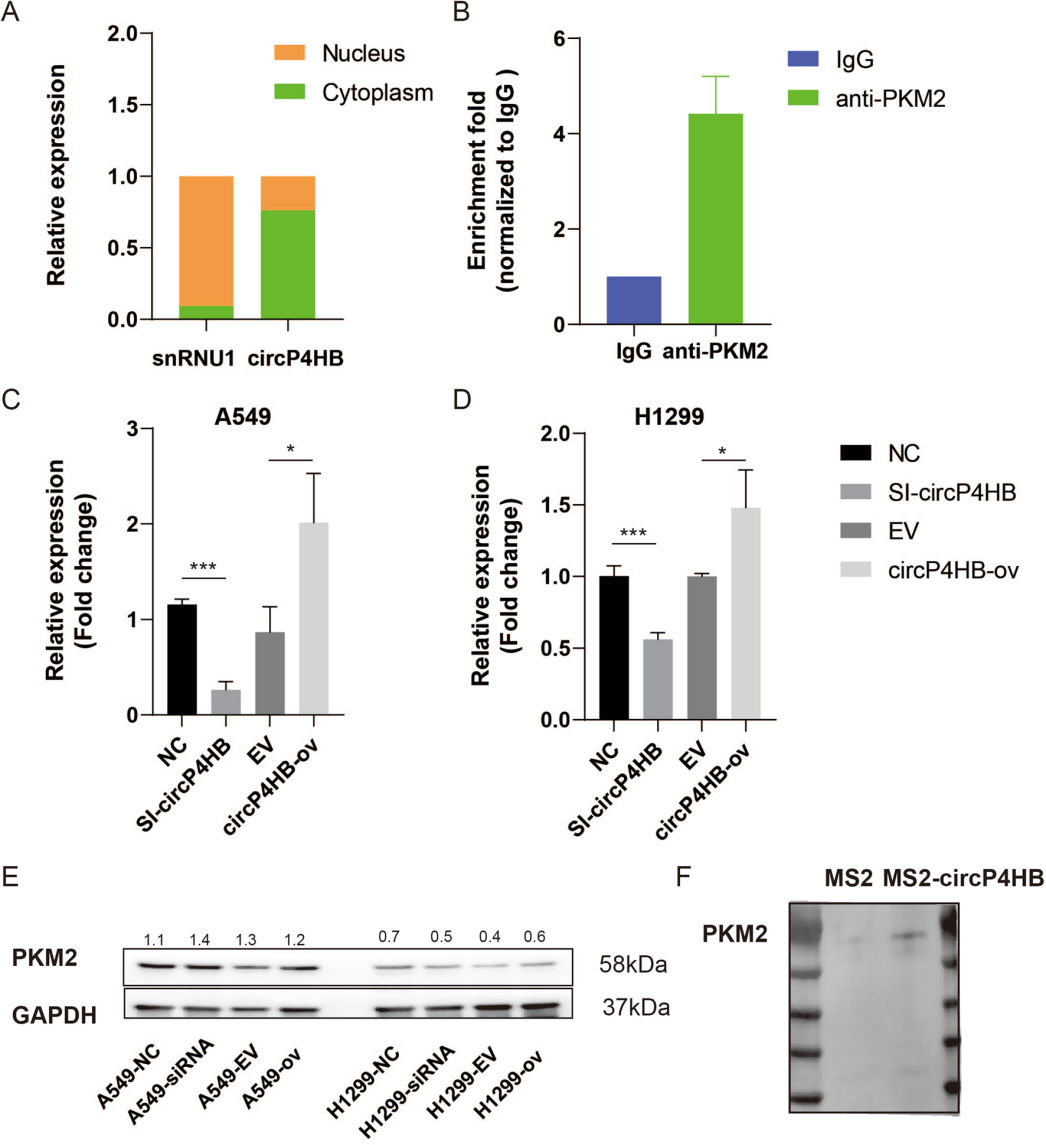
circP4HB can bind to PKM2. **A** circP4HB is mainly located in the cytoplasm. **B** RIP assay showed circP4HB can bind to circP4HB. CircP4HB can regulate the expression of PKM2 in transcriptional level (**C**, **D**) but protein level (**E**). **F** Western blot confirmed the interaction between PKM2 and circP4HB after pull down (*p < 0.05, ***p < 0.001)

To further explore the underlying mechanisms of circP4HB promoting LUAD progression, we first confirmed the subcellular location of cirP4HB. The results of qPCR showed circP4HB is mainly localized in cytoplasm (Fig. 5A) as snRNU1 was the marker of cell nucleus. circRNAs exert their function by interacting with RNA-binding proteins (RBP) in various human diseases, particularly cancers [17]. For instance, circRHOBTB3 can bind with HuR to promote β-Trcp1-mediated ubiquitination of HuR and to reduce the expression level of the downstream target PTBP1 in colorectal cancer [18]. To identify the potential RBPs that might bind to circP4HB, we performed RNA pull-down assays. Labeled with MS2, a vector expressing circP4HB was established. First, we detected circP4HB expression level in A549 cells with transfecting MS2-circP4HB or MS2 alone plasmids (Additional file 4: Fig.S1C). Moreover, we predicted the secondary structure of MS2-circP4HB (Addtional file 4: Fig.S1D). Then, we pulled down RBPs using beads conjugated with the MS2-binding protein after labeling circP4HB with the MS2 structure. Mass spectrometry showed that thirty-eight proteins pulled down by circP4HB labeled with MS2 and pyruvate kinase M2 isoform (PKM2) drew our attention (Additional file 3: Table S3). Moreover, the results of western blot showed circP4HB can bind to PKM2 (Fig. 5F). PKM2 catalyzes the last and physiologically irreversible step in glycolysis, the conversion of phosphoenolpyruvate to pyruvate through the transfer of a phosphate group to adenosine diphosphate [19]. Many studies demonstrated that PKM2 was overexpressed in various cancers and promoted proliferation and metastasis of tumor cells, including LUAD [20, 21]. Moreover, RIP assay was performed using anti -PKM2 antibody and the results further illustrated circP4HB could bind to PKM2 (Fig. 5B). And we predicted the direct interaction between circP4HB and PKM2 in catRAPID database (Additional file 4: Fig.S1E). Next, we asked the potential mechanism of circP4HB to affect PKM2. Therefore, we found downregulated circP4HB can decrease the expression level of PKM2, whereas upregulated circP4HB could increase the PKM2 expression in transcriptional not protein level (Fig. 5C, E).

### circP4HB promoted aerobic glycolysis in LUAD cells

**Fig. 6:**
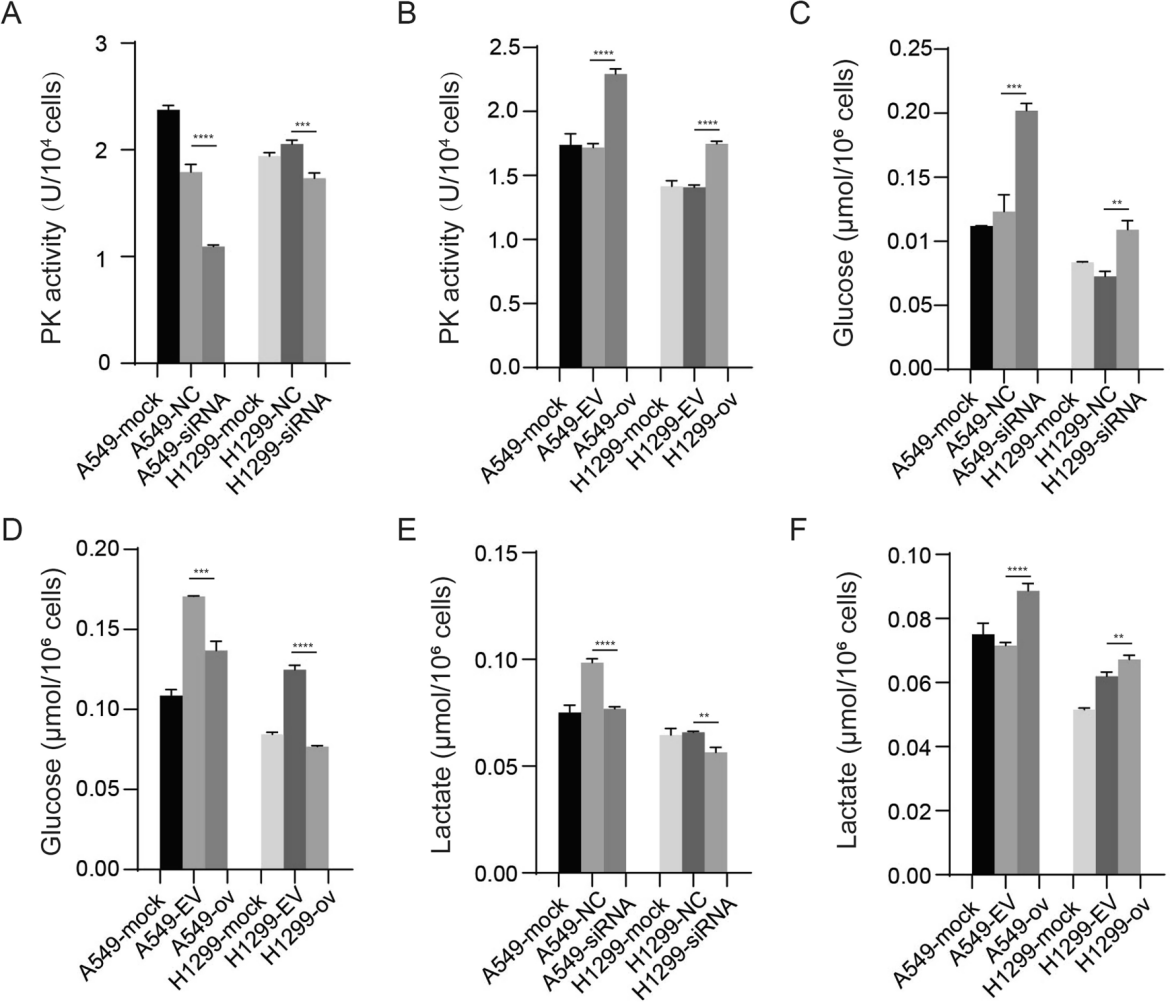
circP4HB could accelerate aerobic glycolysis. CircP4HB can regulate the enzyme activity of PKM2 (**A**, **B**). CircP4HB promoted glucose consumption and lactate production (**C**–**F**). (*p < 0.05, **p < 0.01, ***p < 0.001,****p < 0.0001)

Since PKM2 could catalyze the conversion of phosphoenolpyruvate to pyruvate as a key enzyme, we asked whether circP4HB could influence the enzyme activity of PKM2. Therefore, we evaluated PKM2 activity using manufacture’s activity kit. The results showed circP4HB could increase PKM2 activity (Fig. 6A, B). Then, we raised another question about whether circP4HB could affect the metabolic phenotype in LUAD cells. Glucose consumption and lactate production were detected in the cells, respectively. Results showed that down-regulated circP4HB could decrease glucose consumption and lactate production (Fig. 6C and E). However, this effect of circP4HB on aerobic glycolysis could be reversed when circP4HB was overexpressed (Fig. 6D and F). Taken together, these results above demonstrated circP4HB can increase the enzyme activity of PKM2 and accelerate aerobic glycolysis in LUAD cells.

**Fig. 7:**
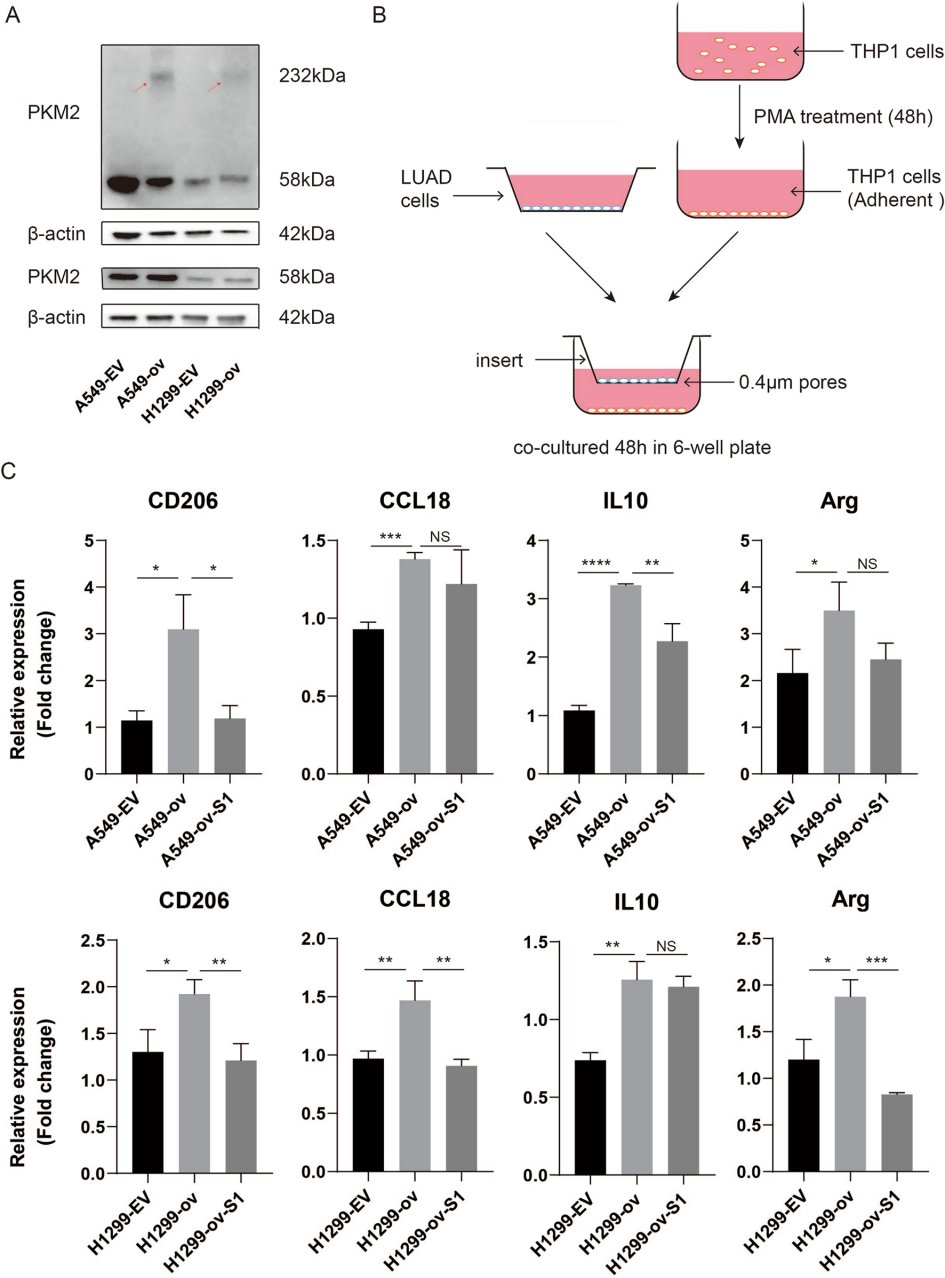
circP4HB promotes M2 macrophage polarization through PKM2. **A** The results of western bolt showed circP4HB can significantly increase the formation of tetrameric PKM2. In vivo crosslinking was performed by incubating the cells with 1 mM disuccinimidyl suberate (DSS) at room temperature for 30 min **B** the schematic diagram of co-culture between THP1 cells and LUAD cells. **C** the results of M2 macrophage markers (CD206/CCL18/IL10/Arg). (*p < 0.05, **p < 0.01, ***p < 0.001, ****p < 0.0001, NS, no significance)

Previous study reported HULC could directly binds with PKM2 and down-regulated its enzymatic activity by promoting PKM2 phosphorylation and inhibiting its tetramer formation [22]. Therefore, we used in vivo crosslinking assay to detect the impact of circP4HB expression on PKM2 tetramer formation. The upregulation of circP4HB did not affect the expression of PKM2, but in vivo crosslinking assay revealed that the overexpression of circP4HB can significantly increase the formation of tetrameric PKM2 (Fig. 7A). But there is no significant difference for phosphate-PKM2 in different groups (Additional file 5: Fig.S2). These results revealed that circP4HB directly binds with PKM2 and upregulates PKM2 enzymatic activity by increasing its tetramer formation.

### circP4HB promoted M2 macrophage polarization

A previous study reported Annexin A5 is identified with the special effect on hepatic macrophage phenotype shift from M1 to M2 by directly interacting with PKM2 [23]. Moreover, Tan et al. showed NFATc1 induced M2 macrophage polarization via by targeting c-myc/PKM2 in cervical cancer [24]. Thus, we asked whether circP4HB can induce M2 macrophage polarization via targeting PKM2. In order to verify our hypothesis, we co-cultured LUAD cells and THP1 cells in 6-well plate with 0.4 μm insert (Fig. 7B). To further verify whether circP4HB affected the LUAD progression via regulating PKM2, we designed siRNAs targeting PKM2 (Additional file 1: Table S1). The results of qPCR and western blot showed the silencing efficiency of siRNAs (Additional file 6: Fig.S3). We detected the markers expression of M2 macrophage, and found that CD206, CCL18, IL10 and Arg significantly increased when circP4HB was overexpressed. Moreover, when PKM2 expression was knockdown, these markers of M2 macrophage were reversed (Fig. 7C). In summary, our results demonstrated that circP4HB can promote M2 macrophage phenotype shift via targeting PKM2.

### circP4HB promoted LUAD progression via binding to PKM2

**Fig. 8:**
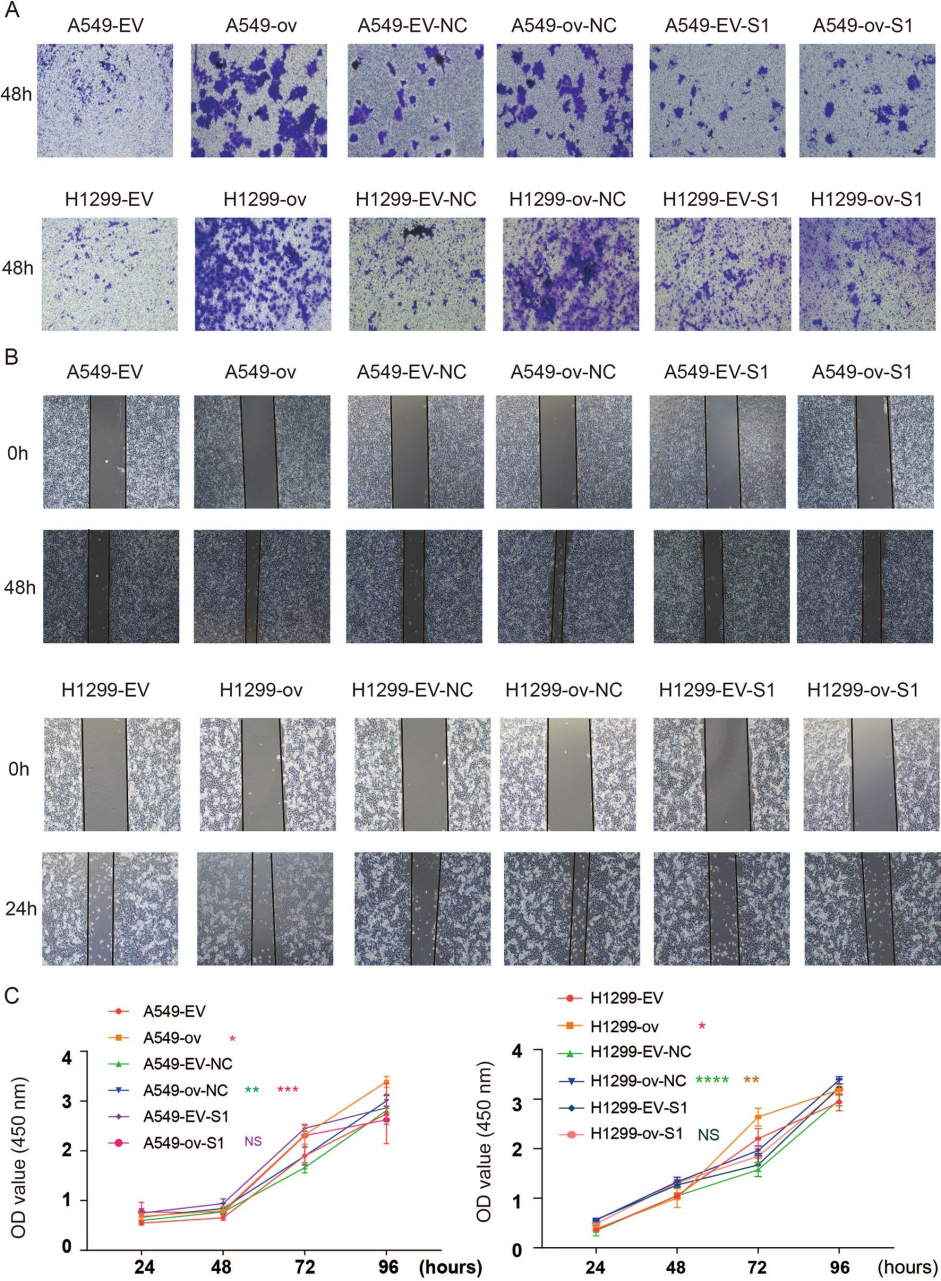
circP4HB promotes LUAD progression by binding PKM2. **A** Transwell showed the invasion ability of LUAD cells. **B** Representative photos of the wound healing assays. After the indicated treatment, images of A549 and H1299 cells were taken at 0 h and 24 h or 48 h after scratching. **C** Cell proliferation was determined by CCK8 assay in A549 and H1299. (*p < 0.05, **p < 0.01, ***p < 0.001, ****p < 0.0001)

circP4HB overexpression increased the invasion ability in A549 and H1299 cells but si-PKM2 can reverse this phenomenon (Fig. 8A, Additional file 7: Fig.S4A-B). Then, wound healing assays showed increased expression of circP4HB promoted the migration of A549 and H1299 cells but knockdown PKM2 can reduce this phenomenon (Fig. 8B, Additional file 7: Fig.S4C-D). As shown by CCK-8 assay results (Fig. 8C), over-expressed circP4HB in A549 and H1299 cells could stimulate cancer cell proliferation, but the proliferative advantage was compromised by knockdown PKM2. Moreover, the colonies formation assays showed similar results with CCK8 (Additional file 8: Fig.S5). This evidence suggested that circP4HB could promote LUAD cell progression via binding to PKM2.

## Discussion

The dysregulation of circRNAs has been increasingly recognized as a common feature in almost all cancers, playing vital roles in the cancer initiation and development, either as tumor suppressors or oncogenes [25]. Through various mechanisms, circRNAs have been shown to regulate cell proliferation, migration, invasion, and other critical processes in different cancer types [26–28]. For example, hsa_circ_0000190 was found to be overexpressed in NSCLC cell lines and promotes tumor cell growth and migration by activating MAPK/ERK pathway [29]. In the present study, we identified a novel circRNA, circP4HB, that promotes LUAD cells proliferation, invasion and migration in vitro and increases tumor growth in vivo. Moreover, we found that circP4HB expression correlates with advanced TNM stage and lymph node metastasis, and LUAD patients with higher circP4HB expression experienced a poorer prognosis. Therefore, we advised that circP4HB may serve as a promising therapeutic target.

Mechanistically, we demonstrated that circP4HB mainly predominantly localizes in the cytoplasm and can bind to PKM2 by RNA pull down and RIP assays. Furthermore, circP4HB regulates PKM2 expression at the transcriptional level and enhances the enzyme activity of PKM2. To our knowledge, it’s first time to report that circP4HB could bind to PKM2 and could increase the PKM2 activity. However, there were some studies reported that lncRNAs can interact with PKM2. For instance, LncRNA HITT physically interacts with PKM2 mapped to a region that has been involved in both dimer and tetramer formation, inhibiting PKM2 oligomerization and leading to dramatic reduction of PKM2 activity [30]. LncRNA GACAT2 overexpression increased the cellular protein expression of PKM1/2, the PKM2 tetramer and phosphorylated PKM2, which led to enhanced pyruvate kinase activity and increased translocation of PKM2 into mitochondria [31]. These evidences illustrated the relationship between aerobic glycolysis and circRNA expression since PKM2 is the key enzyme of glycolysis to transfer phosphoenolpyruvic acid into pyruvate. Indeed, we found upregulated circP4HB could increase lactate production and glucose consumption. As we know, cancer cells produce sufficient energy to meet the growth need by aerobic glycolysis [32]. Several studies have demonstrated circRNAs can promote or disrupt cancer cell development and aerobic glycolysis through different mechanisms [33–35]. circSLC25A16 interacts with miR-488-3p/HIF-1α and activates LDHA by facilitating its transcription and further contributes on glycolysis in NSCLC cells [35]. Then, we found that circP4HB promotes the formation of PKM2 tetramer as the tetramer is the active status of enzyme activity [36]. Numerous studies have shown that the tetramer formation of PKM2 was regulated by post-translational modifications and oncogenic protein or metabolite regulation [37]. Wang et al. suggested that the tetramer formation of PKM2 is affected by its phosphorylation of the Y105 [22], however, we did not observe that change. And previous study demonstrated that lactylation modification can also modulate the enzymatic activity of PKM2 [37]. Furthermore, the tetramer to dimer ratio of PKM2 is not a fixed value and may oscillate depending upon the presence of oncoproteins as well as key metabolic intermediates such as glycerate 3-P [38]. Therefore, the exact mechanism of circP4HB increase the formation of tetrameric PKM2 need to be further explored. Thus, we illustrated circP4HB can induce PKM2 tetramer status and up-regulate PKM2 expression level in translational level to increase the enzyme activity and subsequently promote aerobic glycolysis.

Tumor-associated macrophages (TAMs) polarization often regulated by metabolism such as glycolysis as response to various cytokine stimulations [39]. Moreover, M2 macrophages usually exert a tumor-promoting function whereas M1 macrophages elicit a tumor-inhibiting function in the tumor microenvironment (TME) [40]. Given the roles of M2 macrophage, plenty of strategies have developed to defeat the effects of these cells, which can be divided into two types: reducing the number of TAMs or altering their functionality within the TME [41]. There are a lot of clinical trials have targeted macrophage to improve therapy efficiency, including CSF1/CSF1R agonists, CXCR4 antagonist, CCL2–CCR2 signaling, TLRs and CD40 Agonists and so on [42, 43]. In this study, we found circP4HB can induce THP1 cells into M2 macrophage by co-culturing with LUAD cells. In our knowledge, M2 macrophage in TME is a factor to promote cancer progression, thus, these results further illustrated circP4HB can be an oncogene in LUAD. Moreover, this effect can be reduced when PKM2 was knocked down, which is consistent with the previous report [44]. Taken together, we showed circP4HB can induce M2 macrophage polarization by interacting with PKM2.

In summary, our study identified a novel circRNA, circP4HB, generating from exon 3 and exon 4 of the P4HB gene. CirP4HB could promote LUAD proliferation, migration and invasion in vitro and tumor growth in vivo. Moreover, we disclosed that circP4HB can bind to PKM2 and increase its the enzyme activity by accelerating tetramer formation. Furthermore, circP4HB-PKM2 can accelerate aerobic glycolysis and induce the M2 macrophage polarization in TME. Finally, higher expression of circP4HB is associated with shorter OS and circP4HB might serve as a therapeutic target for LUAD.

### Supplementary Information

**Additional file 1: Table S1.** PCR primers and siRNA sequences.

**Additional file 2: Table S2.** Univariate and multivariate analysis of factors associated with OS.

**Additional file 3: Table S3.** Mass spectrometry results of MS2 pull down.

**Additional file 4: Figure S1.** (A) Quantification results of relative migration distance of Fig. 3G. (B) the results of expression level of circP4HB in A549 after overexpression of circP4HB with or without RNase R digestion. (C) relative expression level of circP4HB in A549 after MS2 or MS2-circP4HB plasmid was transfected into cells. (D) the prediction of secondary structure of MS2-circP4HB in RNAfold web server. (E) the prediction of interaction between circP4HB and PKM2 in catRAPID database. (*p < 0.05, ***p < 0.001, ****p < 0.0001)

**Additional file 5: Figure S2.** The results of western blot of p-pkm2.

**Additional file 6: Figure S3.** The results of qPCR and western blot showed the silencing efficiency of siRNAs of pyruvate kinase M2. (**p < 0.01)

**Additional file 7: Figure S4.** The analysis of cell numbers in Transwell and migration distance of the wound healing assays. (*p < 0.05, **p < 0.01, ***p < 0.001, ****p < 0.0001, NS, no significance)

**Additional file 8: Figure S5.** circP4HB promotes LUAD progression by binding PKM2. Cell proliferation was determined by colony formation assay in A549 (A) and H1299 (B). (**p < 0.01, ****p < 0.000, NS, no significance)

## Data Availability

The datasets generated and/or analyzed during the current study are available from the corresponding author on reasonable request.

## Abbreviations

LC: Lung cancer
NSCLC: Non-small cell lung cancer
LUAD: Lung adenocarcinoma
RBP: RNA binding proteins
PKM2: Pyruvate kinase M2 isoform
PVDF: Polyvinylidene fluoride
TBST: Tris-buffered saline-Tween 20
PMA: Phorbol 12-myristate 13-acetate
CNV: Copy number variation
EV: Empty vector
TAMs: Tumor-associated macrophages
TME: Tumor microenvironment
