# Supplementary material for: Circular RNA P4HB promotes glycolysis and tumor progression by binding with PKM2 in lung adenocarcinoma
Source: Respir Res. 2023 Oct 25;24:252. doi: 10.1186/s12931-023-02563-7 (PMC10601333; doi:10.1186/s12931-023-02563-7)
Supplement: Supplementary file 1 — Additional file 1: Table S1. PCR primers and siRNA sequences. [file 12931_2023_2563_MOESM1_ESM.docx]

TableS1. PCR primers and siRNA sequences

| Genes | Chain | Sequence (5’-3’) |
| --- | --- | --- |
| CircP4HB | Forward | GGCTTCTTCAAGGACGTGGA |
|  | Reverse | GCCTCTCTGCCAGCTTCTTA |
| P4HB-RNA | Forward | CGTGAACTGGCTGAAGAAGC |
|  | Reverse | TGCCTGCAAAAACTGCTTGG |
| P4HB-DNA(CNV) | Forward | GGCGTTGCTCTCCTTATCGT |
|  | Reverse | ACAGCTGAATGCGAAGACCA |
| WSB1-DNA(CNV) | Forward | AAGGCACTGCCACAGATCAT |
|  | Reverse | AGTCACTGCTGAACCAAGCA |
| Arg | Forward | CTTAAAGAACAAGAGTGTGATGTGA |
|  | Reverse | CATGGCCAGAGATGCTTCCA |
| CD206 | Forward | CTGTGGTGCTGTGCATTTATCT |
|  | Reverse | TGAATTGTACTGGTCTGTCCT |
| Il-10 | Forward | CTGGGTCTTGGTTCTCAGCTT |
|  | Reverse | TTAAGGGTTACCTGGGTTGC |
| CCL18 | Forward | TTTCTGGACCCACTTCTTATTG |
|  | Reverse | TCTATACCTCCTGGCAGATTC |
| PKM2 | Forward | TCGCATGCAGCACCTGATT |
|  | Reverse | CCTCGAATAGCTGCAAGTGGTA |
| GAPDH | Forward | CGCTCTCTGCTCCTCCTGTTC |
|  | Reverse | ATCCGTTGACTCCGACCTTCAC |
| si-circP4HB | Sense (5’-3’) | CUCUUUAAGAAGCUGGCAGTT |
|  | Antisense (5’-3’) | CUGCCAGCUUCUUAAAGAGTT |
| PKM2-S1 | Sense (5’-3’) | GCAGCAAGAUCUACGUGGATT |
|  | Antisense (5’-3’) | UCCACGUAGAUCUUGCUGCTT |
| PKM2-S2 | Sense (5’-3’) | CGAUCAGUGGAGACGUUGATT |
|  | Antisense (5’-3’) | UCAACGUCUCCACUGAUCGTT |
| PKM2-S3 | Sense (5’-3’) | GGGUCGAGCAGGAUGUUGATT |
|  | Antisense (5’-3’) | UCAACAUCCUGCUCGACCCTT |
| Negative control | Sense (5’-3’) | UUCUCCGAACGUGUCACGUTT |
|  | Antisense (5’-3’) | ACGUGACACGUUCGGAGAATT |
