# Supplementary figures and images for: Circular RNA P4HB promotes glycolysis and tumor progression by binding with PKM2 in lung adenocarcinoma
Source: Respir Res. 2023 Oct 25;24:252. doi: 10.1186/s12931-023-02563-7 (PMC10601333; doi:10.1186/s12931-023-02563-7)

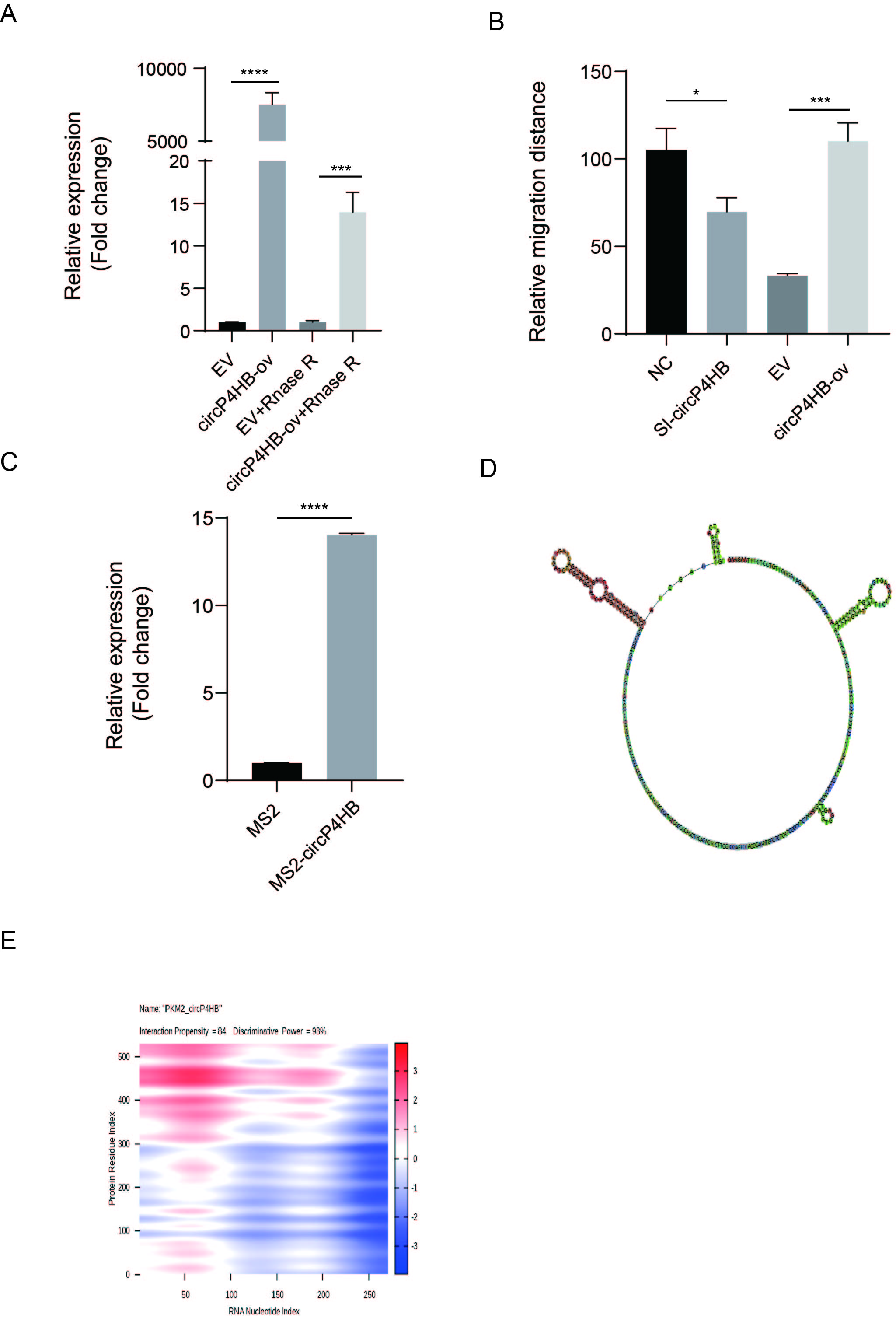

Supplement: Supplementary file 4 — Additional file 4: Figure S1. (A) Quantification results of relative migration distance of Fig. 3G. (B) the results of expression level of circP4HB in A549 after overexpression of circP4HB with or without RNase R digestion. (C) relative expression level of circP4HB in A549 after MS2 or MS2-circP4HB plasmid was transfected into cells. (D) the prediction of secondary structure of MS2-circP4HB in RNAfold web server. (E) the prediction of interaction between circP4HB and PKM2 in catRAPID database. (*p < 0.05, ***p < 0.001, ****p < 0.0001) [file 12931_2023_2563_MOESM4_ESM.jpg]

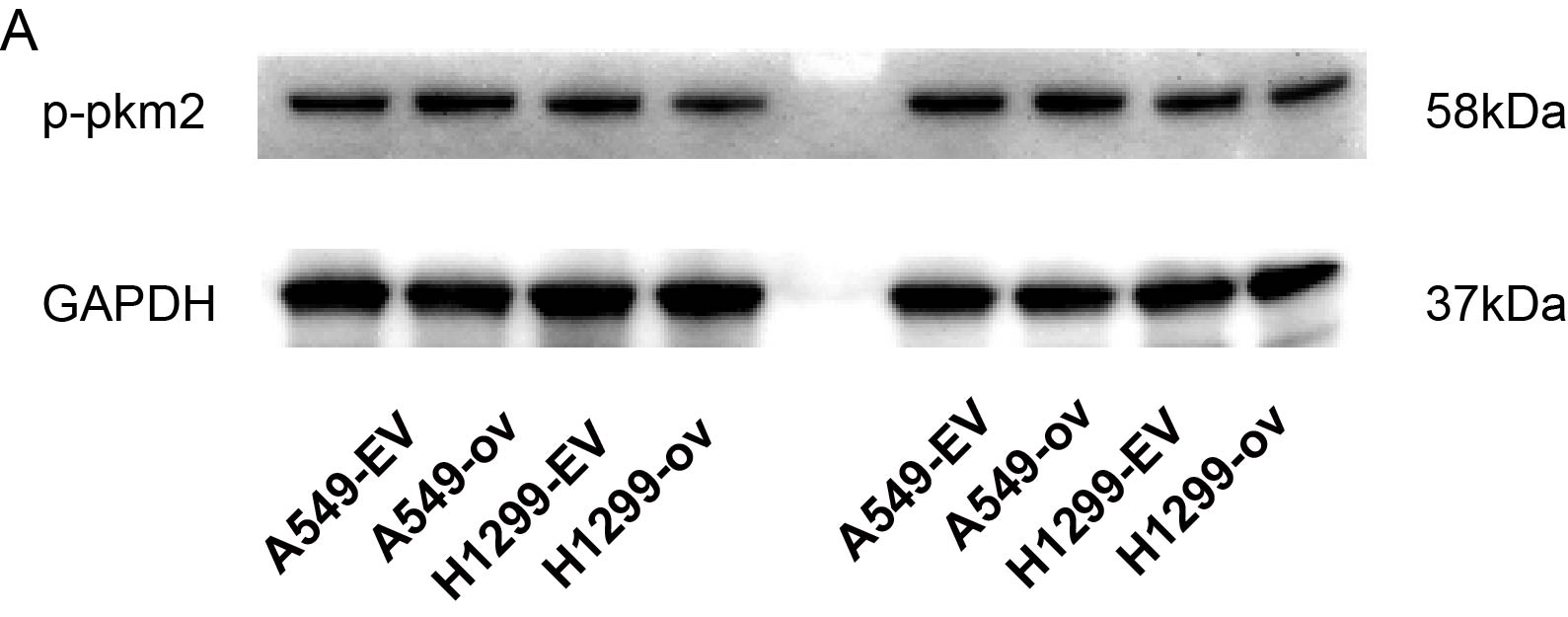

Supplement: Supplementary file 5 — Additional file 5: Figure S2. The results of western blot of p-pkm2. [file 12931_2023_2563_MOESM5_ESM.jpg]

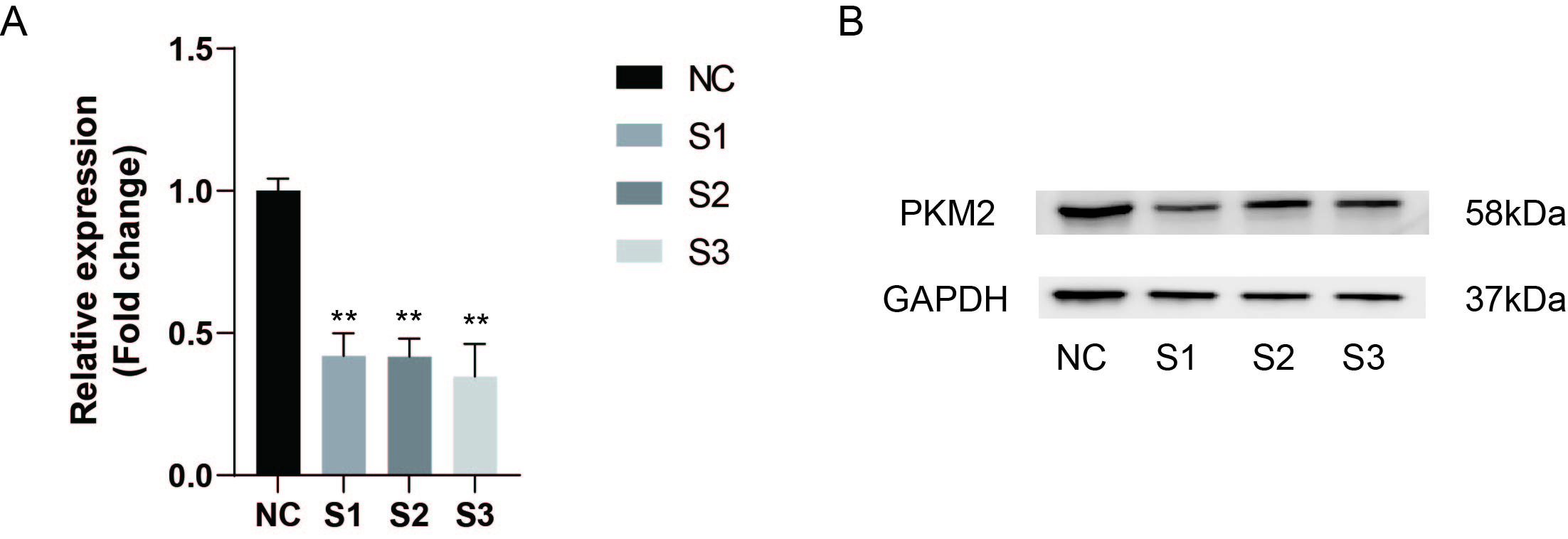

Supplement: Supplementary file 6 — Additional file 6: Figure S3. The results of qPCR and western blot showed the silencing efficiency of siRNAs of pyruvate kinase M2. (**p < 0.01) [file 12931_2023_2563_MOESM6_ESM.jpg]

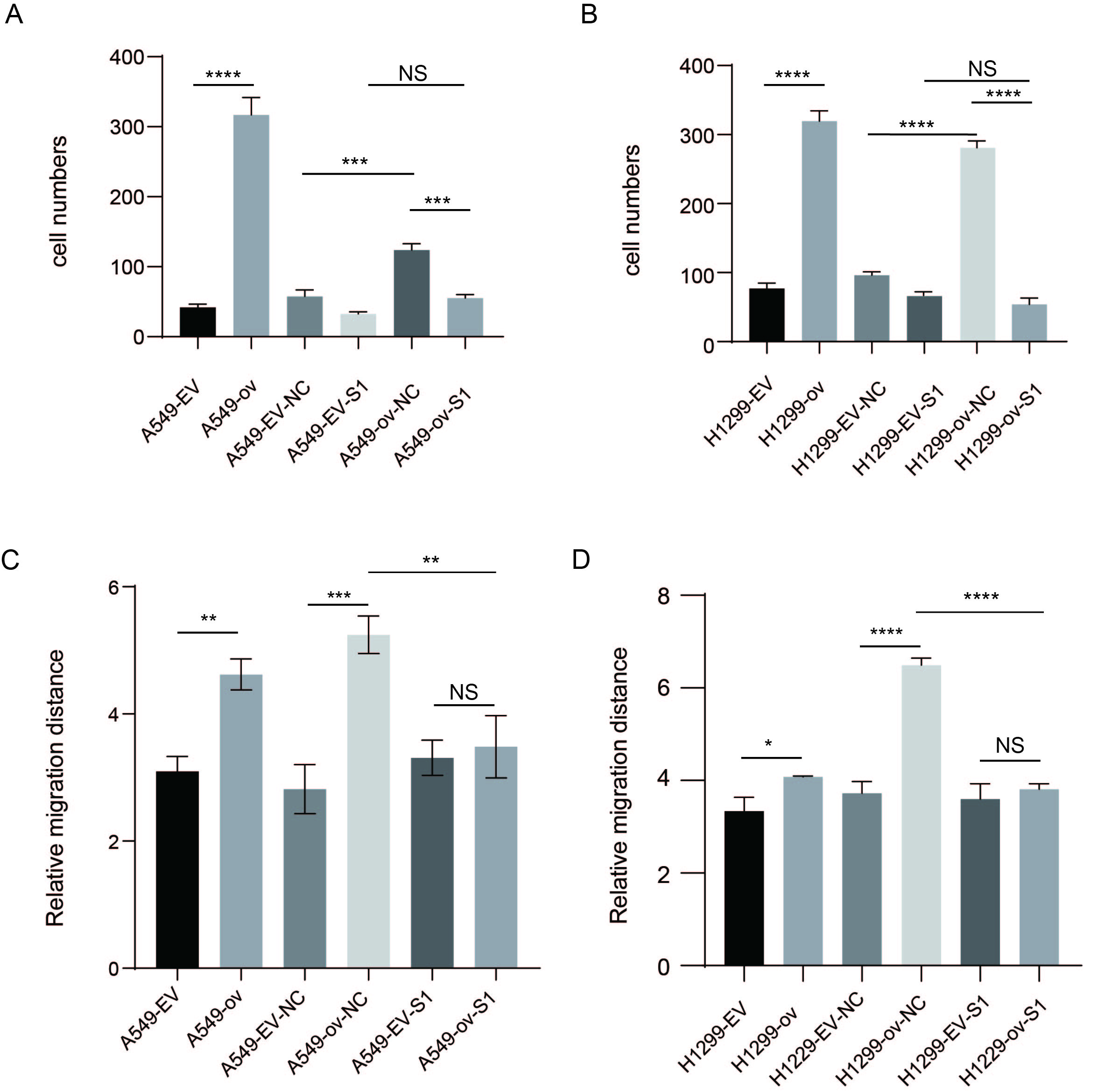

Supplement: Supplementary file 7 — Additional file 7: Figure S4. The analysis of cell numbers in Transwell and migration distance of the wound healing assays. (*p < 0.05, **p < 0.01, ***p < 0.001, ****p < 0.0001, NS, no significance) [file 12931_2023_2563_MOESM7_ESM.jpg]

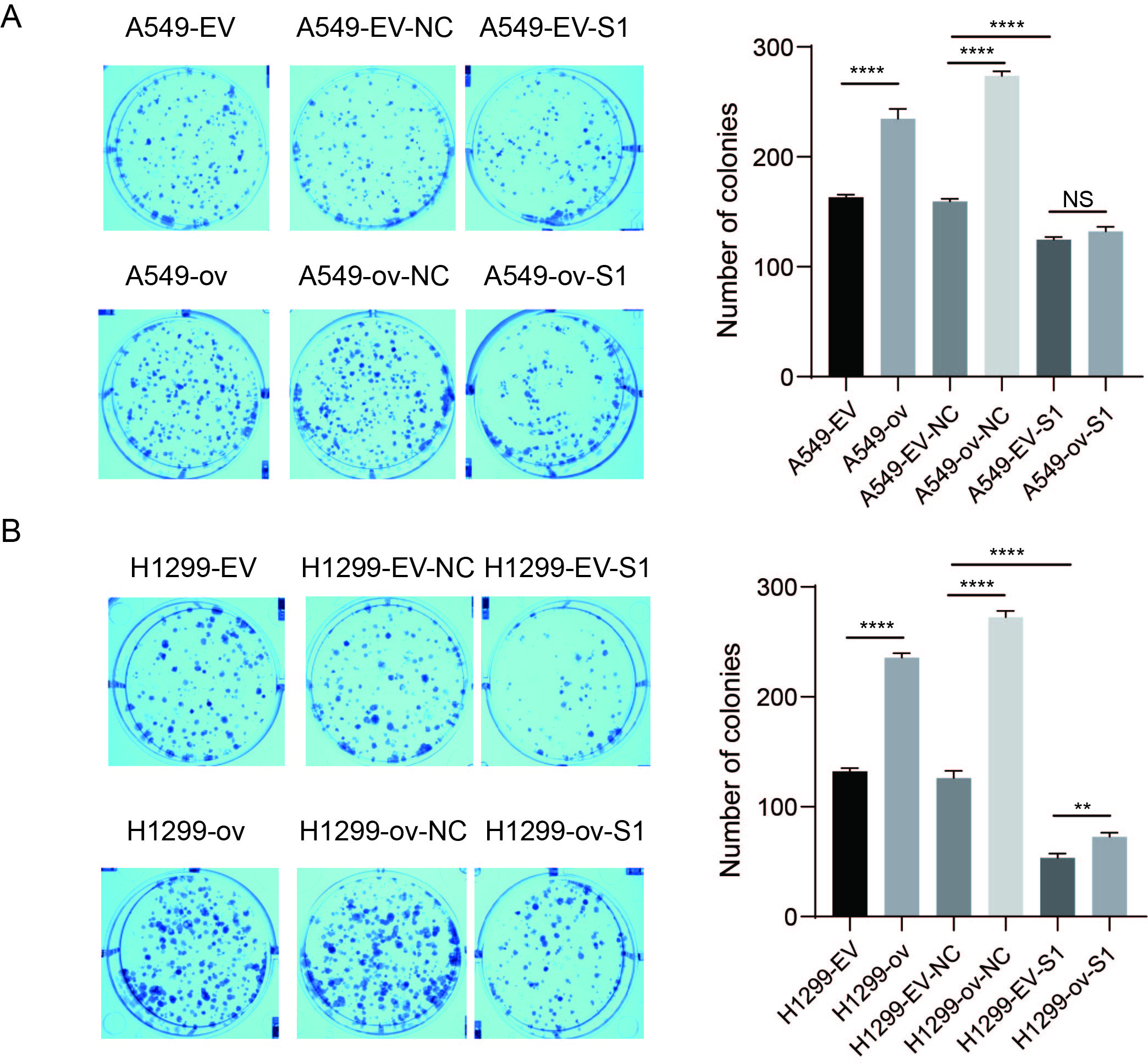

Supplement: Supplementary file 8 — Additional file 8: Figure S5. circP4HB promotes LUAD progression by binding PKM2. Cell proliferation was determined by colony formation assay in A549 (A) and H1299 (B). (**p < 0.01, ****p < 0.000, NS, no significance) [file 12931_2023_2563_MOESM8_ESM.jpg]
